# 618. Evaluation of the Burden of Injection Drug Use-Related Infections in Tennessee

**DOI:** 10.1093/ofid/ofaf695.191

**Published:** 2026-01-11

**Authors:** Emily Moore, Anastasia Cajigal, Christine M Thomas, Amber J Coyne

**Affiliations:** Vanderbilt University Medical Center, Nashville, Tennessee; Tennessee Department of Health, Nashville, Tennessee; Tennessee Department of Health, Nashville, Tennessee; Tennessee Department of Health, Nashville, Tennessee

## Abstract

**Background:**

Injection-related infections (IRIs) in people with substance use disorder (SUD) are associated with non-sterile injection drug use (IDU) practices. IRIs are associated with high rates of hospitalization and high episodic costs of care, but limited surveillance impedes understanding of their impact. Describing the burden and cost of IRIs can inform efforts to mitigate the syndemic of SUD and associated conditions.

**Figure:**
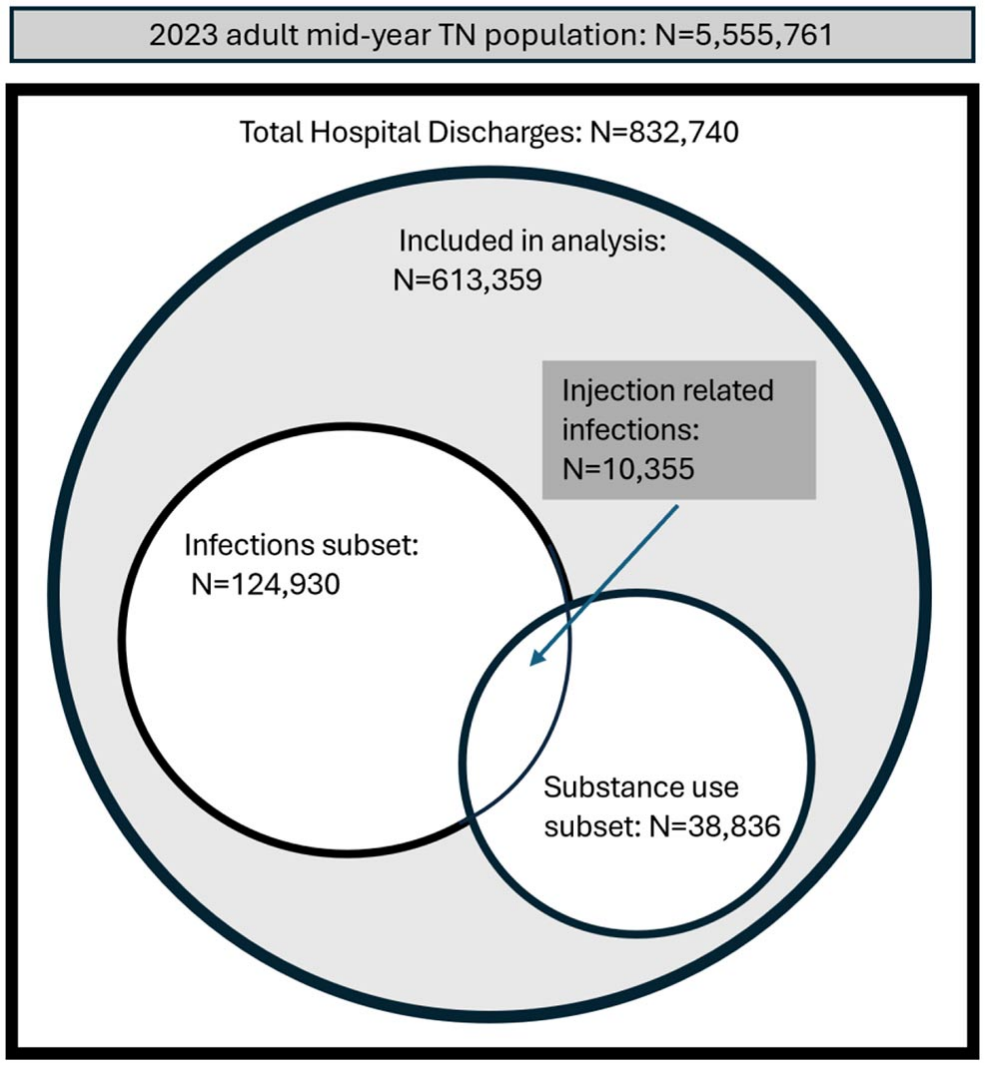
Tennessee hospitalizations in 2023

**Figure:**
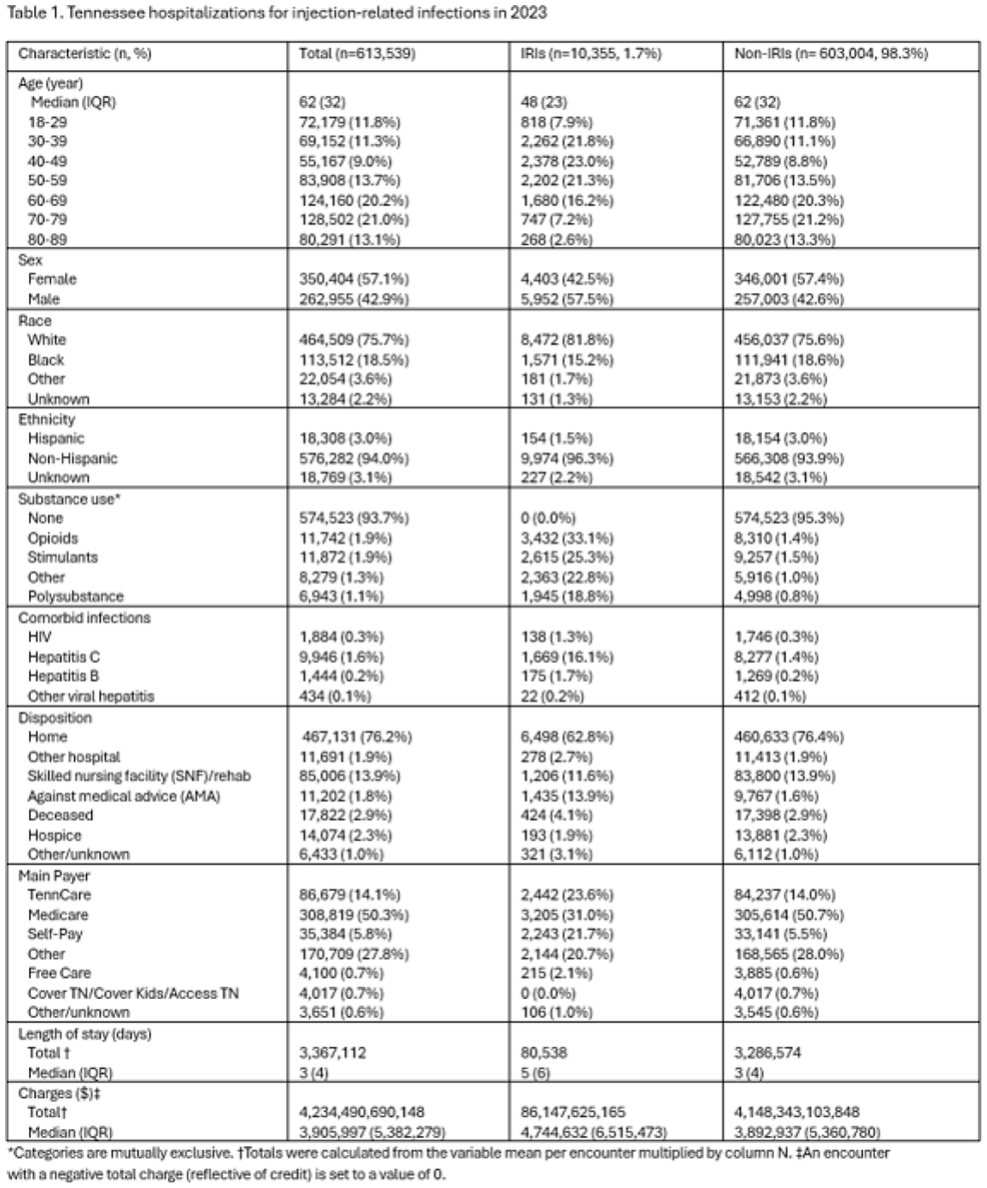

**Methods:**

We conducted a cross-sectional study of Tennessee (TN) residents aged 18-89 years old discharged from a TN acute-care or rehab hospital from 1/1-12/31/2023, using the TN Hospital Discharge Data System, to compare demographics, hospital charges, and primary payer between IRI vs. non-IRI encounters. A discharge met criteria for an IRI if it had ICD-10 codes for both substance use (e.g. opioids), and an acute IDU-associated infection (e.g. endocarditis). We excluded records with missing age, sex, and ICD-10 codes. We described categorical variables with counts (%) and continuous variables with median (IQR). Cumulative incidence was calculated using 2023 mid-year TN census data. Data was analyzed with R and Stata v.18. The project was approved by the TN Department of Health Institutional Review Board.

**Figure:**
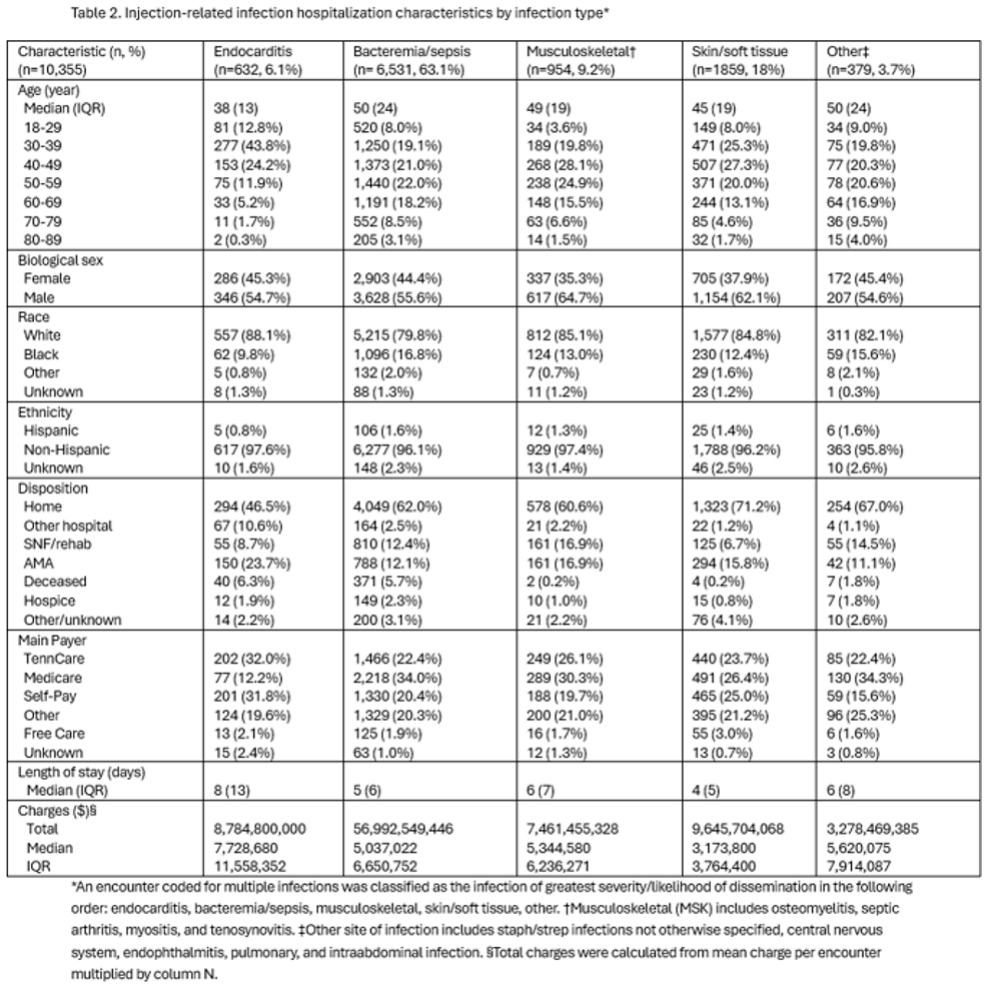

**Results:**

There were 613,539 hospital discharge records included in analysis. IRIs requiring hospitalization numbered 10,355 (1.7%), with a cumulative incidence of 186 cases per 100,000 people. Compared with the non-IRI group (n=603,004; 98.3%), IRI patients were more frequently younger (age 48 [IQR=23] vs 62 [IQR=32]) and had a main payer of “Self-pay” (n=2,243; 21.7% vs n=33,141; 5.5%) or TennCare (TN Medicaid; n=2,442; 23.6% vs n=84,237; 14.0%). IRIs incurred $86,147,625,165 in charges, which was 2.0% of total hospitalization costs in TN. IRIs were linked to 80,538 in-hospital days and 617 (1.9%) of in-hospital deaths and hospice discharges.

**Conclusion:**

IRIs are a significant burden to patients and health systems. Syndemic program planning should consider potential savings to public insurance and uncompensated care programs through mitigation of IRIs. Information about the distribution of IRIs can inform placement of harm reduction services in areas at high risk of IRIs.

**Disclosures:**

All Authors: No reported disclosures

